# Efficacy and Safety of Qiwei Tongbi Oral Liquid in Patients with Stable Long-Standing Rheumatoid Arthritis

**DOI:** 10.1155/2021/3930800

**Published:** 2021-10-23

**Authors:** Wenjun Lu, Liping Fang, Jijie Zhang

**Affiliations:** ^1^Department of Rheumatology and Immunology, The People's Hospital of Danyang, Affiliated Danyang Hospital of Nantong University, China; ^2^Traditional Chinese Medicine Department, The People's Hospital of Danyang, Affiliated Danyang Hospital of Nantong University, China

## Abstract

**Objective:**

Our study is aimed at investigating the efficacy and safety of Qiwei Tongbi oral liquid in patients with stable long-standing rheumatoid arthritis (RA).

**Method:**

140 patients with stable long-standing RA were recruited into the Qiwei Tongbi oral liquid group or the control group. At study recruitment and after 12 weeks of treatment, their C-reactive protein (CRP) levels, interleukin-6 (IL-6) levels, erythrocyte sedimentation rate (ESR), Health Assessment Questionnaire (HAQ), visual analogue scale (VAS), and Disease Activity Score (DAS) 28 were compared in two groups.

**Results:**

Patients in the Qiwei Tongbi oral liquid group had a lower level of CRP, IL-6, VAS scale, and HAQ score compared to patients in the control group (CRP: 3.51 ± 1.57 vs.5.47 ± 1.72 mg/L, *P* < 0.001; IL-6: 1.62 ± 0.8 vs. 2.19 ± 0.88 pg/mL, *P* < 0.001; VAS scale: 1.59 ± 0.69 vs. 2.66 ± 1.02, *P* < 0.001; and HAQ score: 1.19 ± 0.46 vs. 1.41 ± 0.50, *P* = 0.005). The ESR and DAS28 did not reach statistical difference. No damage to liver and kidney functions was observed in both groups.

**Conclusion:**

Qiwei Tongbi oral liquid has the tendency to decrease the inflammation levels and pain score and improve patients' outcomes in patients with stable long-standing RA.

## 1. Introduction

Rheumatoid arthritis (RA) is one of the most common autoimmune diseases and causes 10 to 15 years of life expectancy shorter than the general population. Patients with RA are more likely to die early and live with more complications, such as cardiovascular problems and respiratory diseases [1]. RA causes systematic inflammation, which leads to destruction in the joints. It also causes extra-articular manifestations [2]. It is well acknowledged that RA should be treated at an early stage with all treatments targeted at the suppression of inflammation as it helps to control the disease, decrease disease progression, and raise patients' quality of life and physical function [3]. However, less attention is paid to patients with stable disease. Most of the previously published clinical studies recruited only patients in the active disease. Patients in the active stage or early stage are suggested to treat with traditional disease-modifying antirheumatic drugs (DMARDs), which comprise a wide range of drugs treated for RA to slow down the disease progress. Only a small number of clinical studies evaluated the treatment of patients with stable long-standing RA. A multicenter randomized controlled trial (RCT) of 466 patients with more than 5 years of RA with stable disease of at least 6 months suggested that patients with DMARDs gained no additional benefit in inflammation levels, such as C-reactive protein (CRP) levels [4]. However, it is still meaningful to give these patients the best consultation and care as their disease may progress without continuous attention.

Traditional Chinese medicine (TCM) has long been used to treat RA since it helped patients suppress the inflammation and raise their immune function [5]. TCM has several active components that could help to relieve inflammation. It can also produce and store immune cells to prevent inflammation [6]. Among these TCMs, Qiwei Tongbi oral liquid has long been used to treat RA through its immune-enhancing function and suppression of inflammation in Asian countries. Its main active components are ants, Sinomenium acutum, Millettia dielsiana, and Pyrola calliantha H. Andres. These components are regarded to have the function to decrease the inflammation levels and increase patients' overall well-being as TCM for thousands of years [7]. However, no previous studies evaluated its use in patients with stable long-standing RA. Thus, we decided to investigate its function in the suppression of inflammation and health status in patients with stable long-standing RA.

This study recruited only patients with at least 5 years of RA disease and at least at the stable stage for 6 months. Patients were randomized into two groups into the Qiwei Tongbi oral liquid or control group. Their inflammation levels, including CRP, interleukin-6 (IL-6), and erythrocyte sedimentation rate (ESR), were measured using questionnaires to assess the activity of RA such as the Health Assessment Questionnaire (HAQ), visual analogue scale (VAS), and Disease Activity Score (DAS) 28; liver and kidney functions were compared between two groups.

Our hypothesis is that Qiwei Tongbi oral liquid could decrease the inflammation levels and maintain a better condition in patients with stable long-standing RA.

## 2. Patients and Methods

### 2.1. Patients

140 patients were recruited with age over 60 years and at least 5 years of RA history and at least with stable disease for 6 months. They were recruited from 2017 to 2019 in Danyang People's Hospital. Patients in the treatment group received Qiwei Tongbi oral liquid 10 mL three times a day after meals. Patients in the control group received control medicine orally with the same smell in the same color, character, and texture three times a day. No DMARD or systematic treatment was given to these patients. Patients in both groups were treated for 3 months. We recorded patients' baseline clinical characteristics, including age, sex, RA disease duration, CRP, IL-6, and ESR; patient-reported questionnaires including HAQ, VAS, and DAS28; and liver and kidney functions at recruitment and 3 months after treatment in two groups. The study was approved by the Ethics Committee of Danyang People's Hospital. All patients signed written informed consent.

### 2.2. Inflammation Level Analysis

Patients' inflammatory biomarkers including CRP, ESR, and IL-6 were measured at treatment recruitment and 3 months after treatment following standard laboratory procedures at the Department of Clinical Chemistry, Danyang People's Hospital.

### 2.3. Liver and Kidney Functions

Patients' liver functions including alanine aminotransferase (ALT), aspartate transaminase (AST), gamma-glutamyl transferase (GGT), alkaline phosphatase, and bilirubin were monitored at baseline and 3 months after treatment in two groups following standard laboratory procedures at the Department of Clinical Chemistry, Danyang People's Hospital. Patients' kidney functions were also monitored. The parameters included creatinine, uric acid, and urea nitrogen.

### 2.4. Patient-Reported Outcomes

Patients were required to do questionnaires to assess their RA status. HAQ is a questionnaire that helps to comprehensively measure the outcome of RA patients [8]. Patients need to answer if they were able to do the dressing and grooming, arising from chair, eating, walking and doing hygiene, etc. VAS is designed to measure the pain caused by RA with 0 meaning no pain and 10 meaning the worst pain imaginable [9]. DAS28 is a measure of disease activity in RA that records swollen and tender joints and laboratory values including CPR and ESR [10]. Patients in two groups were asked to do these three questionnaires at recruitment and 3 months after treatment.

### 2.5. Statistical Analysis

Patient baseline characteristics and their parameters 3 months after treatment, including CRP, ESR, and IL-6 analyses, and HAQ, VAS, and DAS28 were compared using chi-squared tests for categorical variables and Fisher's exact test for continuous variables. Statistical significance was defined as *P* ≤ 0.05. All statistical analysis was performed using SPSS, version 16.0 (IBM Corporation, Armonk, NY, USA).

## 3. Results

### 3.1. Baseline Characteristics

140 patients were recruited in our study. 70 patients were treated with Qiwei Tongbi oral liquid and 70 patients in the control group. Patients in the control group received control medicine with the same color, texture, and smell as Qiwei Tongbi oral liquid. Baseline clinical characteristics, including age, sex, RA disease duration, CRP, IL-6, and ESR, and patient-reported questionnaires including HAQ, VAS, and DAS28 were compared between two groups in Table 1. Patient baseline characteristics between two groups did not have statistical difference (*P* > 0.05). Their liver and kidney functions are normal and did not reach statistically significant differences in the two groups.

### 3.2. Comparisons of CRP, ESR, and IL-6 between Two Groups

**Figure 1:**
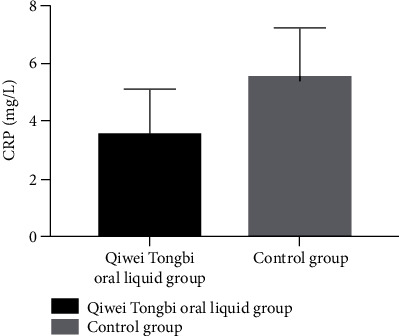
Comparison of CRP between two groups. Patients in the Qiwei Tongbi oral liquid group had lower levels of CRP compared to patients in the control group. Abbreviations: CRP = C-reactive protein.

**Figure 2:**
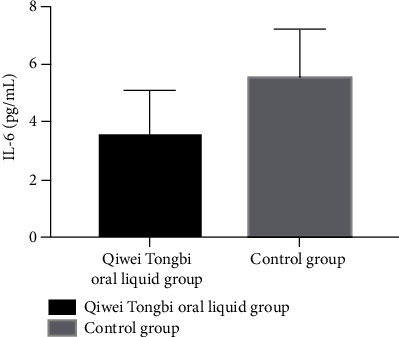
Comparison of IL-6 between two groups. Patients in the Qiwei Tongbi oral liquid group had lower levels of IL-6 compared to patients in the control group. Abbreviations: IL-6 = interleukin-6.

Patient baseline CRP, ESR, and IL-6 did not have statistical differences between two groups (CRP: 3.29 ± 1.31 vs. 3.01 ± 1.17 mg/L, *P* = 0.199; ESR: 22.59 ± 1.26 vs. 22.3 ± 1.83 mm/h, *P* = 0.271; and IL-6: 1.52 ± 0.52 vs. 1.41 ± 0.68, *P* = 0.275, Table 1). Patients in the Qiwei Tongbi oral liquid group had lower levels of CRP and IL-6 compared to patients in the control group after 3 months of treatment (CRP: 3.51 ± 1.57 vs. 5.47 ± 1.72 mg/L, *P* < 0.001, Figure 1; IL-6: 1.62 ± 0.8 vs. 2.19 ± 0.88 pg/mL, *P* < 0.001, Figure 2). The ESR at 12 weeks after treatment between two groups did not reach statistical difference (ESR: 25.81 ± 4.19 vs. 26.67 ± 4.75 mm/h, *P* = 0.256).

### 3.3. Comparison of Patient-Reported Outcomes between Two Groups

**Figure 3:**
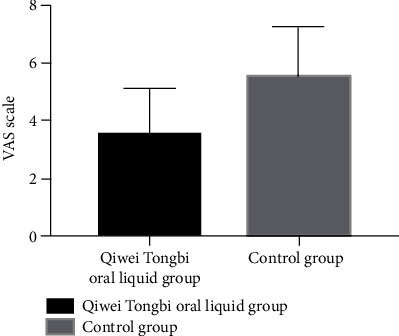
Comparison of VAS between two groups. Patients in the Qiwei Tongbi oral liquid group had lower levels of VAS compared to patients in the control group. Abbreviations: VAS = visual analogue scale.

**Figure 4:**
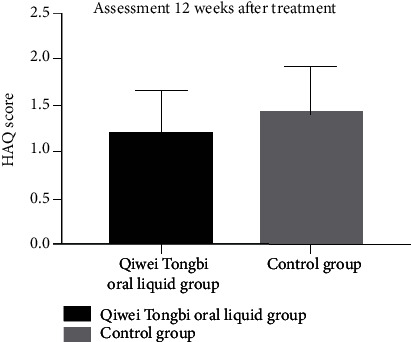
Comparison of HAQ between two groups. Patients in the Qiwei Tongbi oral liquid group had lower levels of HAQ compared to patients in the control group. Abbreviations: HAQ = Health Assessment Questionnaire.

Patients' baseline HAQ, VAS, and DAS28 did not have statistical differences between two groups (HAQ: 1.27 ± 0.45 vs. 1.3 ± 0.46, *P* = 0.711; VAS: 1.49 ± 0.58 vs. 1.67 ± 0.63, *P* = 0.080; and DAS28: 3.5 ± 0.82 vs. 3.42 ± 0.96, *P* = 0.558, Table 1). Patients in the Qiwei Tongbi oral liquid group had a lower level of the VAS scale and HAQ score compared to patients in the control group after 3 months of treatment (VAS scale: 1.59 ± 0.69 vs. 2.66 ± 1.02, *P* < 0.001, Figure 3; HAQ score: 1.19 ± 0.46 vs. 1.41 ± 0.50, *P* = 0.005, Figure 4). The DAS28 at 12 weeks after treatment between two groups did not reach statistical difference (3.74 ± 0.78 vs. 3.76 ± 1.0, *P* = 0.888).

### 3.4. Comparison of Liver and Kidney Functions between Two Groups

Patient baseline liver and kidney functions at baseline and 3 months after treatment were normal and did not have statistical differences between two groups.

## 4. Discussion

Patients with stable long-standing RA are facing risks of deterioration even though DMARDs were given to these patients [11]. Thus, an alternative treatment method is needed to help patients maintain a good health condition. TCM has long been used as a function to control systematic inflammation and increase immune function [12]. However, no previous studies published the impact of Qiwei Tongbi oral liquid in the regulation of inflammation and RA activities in patients with stable long-standing RA. Our study suggested that patients with stable long-standing RA receiving Qiwei Tongbi oral liquid had lower levels of inflammation and better condition of overall status in comparison to patients in the control group.

Qiwei Tongbi oral liquid has been used to treat RA in Asia. It contains seven components that are used to treat swollen joints and release pain. Among them, Sinomenium acutum has an anti-inflammatory effect and an inhibitory effect on lymphocyte proliferation in a rat model [13]. Rats treated with Sinomenium acutum have a lower level of inflammatory parameters, such as tumor necrosis factor-*α* (TNF-*α*) and prostaglandin-E2 (PG-E2) [14]. Millettia dielsiana is a TCM to treat RA and gynecological diseases used as antioxidant, immunomodulatory, and anticoagulant agents [15]. It can help RA patients relieve muscle aches and pains. Pyrola calliantha H. Andres is used as a sedative and an analgesic against RA [16]. Our study was in accordance with previous findings, suggesting that Qiwei Tongbi oral liquid was helpful in suppressing the CRP and IL-6 levels in patients with stable long-standing RA.

RA is an inflammatory disease and has been treated with various TCMs [17]. In a review of TCM, RA is caused by the invasion of wind, dampness, or heat pathogens into the human body [18, 19]. TCMs help patients maintain better overall well-being and relieve swelling of joints and pain. TCM is also useful in alleviating cartilage destruction and other features including synovial hyperplasia in a rat model [20]. A protocol from Shanghai University of Traditional Chinese Medicine indicated that they would like to investigate the impact of one of the TCMs Jia Wei Niu Bang Zi granule in 120 patients with active RA. The hypothesis of their study is that it is beneficial in patients with RA. In their study, DAS was applied to assess the injury and disease severity of RA. Their study had the potential to be the evidence that TCM was effective on life quality, joint function, and symptoms in patients with RA [21]. Another study indicated that TCM was helpful in improving RA-associated fatigue [22]. A study of another TCM Juanbi pill has also been widely used in China for decades to treat RA. A study is ongoing to investigate the combination of Juanbi pill with methotrexate in active RA and hypothesized that TCM is useful in treating active RA [23]. The findings of a meta-analysis with 20 RCTs indicated that TCM could obtain efficacy and safety in the treatment of RA. Using TCM as adjunctive therapy in RA has great treatment benefits for further RA development [24]. Another systematic review and meta-analysis is still ongoing to validate the efficacy and safety of TCM for active RA [25]. Our study focused on patients with stable long-standing RA and investigated the impact of Qiwei Tongbi oral liquid in the patient-reported outcomes including HAQ, VAS, and DAS28. The results suggested that patients receiving Qiwei Tongbi oral liquid had better HAQ and VAS levels than patients in the control group.

However, we admitted that our study has limitations. First, our study recruited only 150 patients. Thus, our results need to be validated in a large-scale, good designed clinical trial. Second, TCM including Qiwei Tongbi oral liquid contains several active components. Its mechanism to be used in patients with RA is still unknown. However, this medicine has been used for decades in Asia, and its effect is well acknowledged by experts. We encourage more basic research about the mechanisms of TCMs so that their efficacy and safety can be better evaluated.

## 5. Conclusions

Qiwei Tongbi oral liquid could help patients with stable long-standing RA relieve inflammation and reach a better overall state.

## Figures and Tables

**Table 1:** Patients' baseline characteristics.

Characteristics	Qiwei Tongbi oral liquid group (*n* = 70)	Control group (*n* = 70)	*P* value
Sex			0.391
Males	25	30	
Females	45	40	
Age (mean age)	67	68	0.196
Rheumatoid arthritis duration (years)	6	6	0.758
CRP (mg/L)	3.29 ± 1.31	3.01 ± 1.17	0.199
ESR (mm/h)	22.59 ± 1.26	22.3 ± 1.83	0.271
IL-6 (pg/mL)	1.52 ± 0.52	1.41 ± 0.68	0.275
HAQ	1.27 ± 0.45	1.3 ± 0.46	0.711
VAS	1.49 ± 0.58	1.67 ± 0.63	0.080
DAS28	3.5 ± 0.82	3.42 ± 0.96	0.558

Mean values of two groups were compared using chi-squared tests for categorical variables and Fisher's exact test for continuous variables. Abbreviations: CRP = C-reactive protein; ESR = erythrocyte sedimentation rate; IL-6 = interleukin-6; HAQ = Health Assessment Questionnaire; VAS = visual analogue scale; DAS = Disease Activity Score.

## Data Availability

Data are available from the corresponding author on reasonable request.
